# Reliability and validity of a non-linear index of heart rate variability to determine intensity thresholds

**DOI:** 10.3389/fphys.2024.1329360

**Published:** 2024-02-05

**Authors:** Noemí Sempere-Ruiz, José Manuel Sarabia, Sabina Baladzhaeva, Manuel Moya-Ramón

**Affiliations:** ^1^ Department of Sport Sciences, Sport Research Centre, Miguel Hernandez University, Elche, Spain; ^2^ Alicante Institute for Health and Biomedical Research (ISABIAL), Alicante, Spain

**Keywords:** intensity domains, graded exercise test, DFA alpha 1, cycle ergometer, power output, oxygen uptake, ventilatory thresholds, lactate thresholds

## Abstract

Exercise intensity distribution is crucial for exercise individualization, prescription, and monitoring. As traditional methods to determine intensity thresholds present limitations, heart rate variability (HRV) using DFA a1 has been proposed as a biomarker for exercise intensity distribution. This index has been associated with ventilatory and lactate thresholds in previous literature. This study aims to assess DFA a1’s reliability and validity in determining intensity thresholds during an incremental cycling test in untrained healthy adults. Sixteen volunteers (13 males and 3 females) performed two identical incremental cycling stage tests at least 1 week apart. First and second ventilatory thresholds, lactate thresholds, and HRV thresholds (DFA a1 values of 0.75 and 0.5 for HRVT1 and HRVT2, respectively) were determined in heart rate (HR), relative oxygen uptake (VO_2_rel), and power output (PO) values for both tests. We used intraclass correlation coefficient (ICC), change in mean, and typical error for the reliability analysis, and paired t-tests, correlation coefficients, ICC, and Bland-Altman analysis to assess the agreement between methods. Regarding reliability, HRV thresholds showed the best ICCs when measured in PO (HRVT1: ICC = .87; HRVT2: ICC = .97), comparable to ventilatory and lactate methods. HRVT1 showed the strongest agreement with LA 2.5 in PO (*p* = 0.09, *r* = .93, ICC = .93, bias = 9.9 ± 21.1), while HRVT2 reported it with VT2 in PO (*p* = 0.367, *r* = .92, ICC = .92, bias = 5.3 ± 21.9). DFA a1 method using 0.75 and 0.5 values is reliable and valid to determine HRV thresholds in this population, especially in PO values.

## 1 Introduction

Exercise intensity distribution is an important factor to individualize, prescribe, and monitor exercise training programs in performance (Bourgois et al., 2019) and clinical (Mezzani et al., 2012) populations. The determination of physiologic breakpoints during endurance exercise is essential for intensity domain classification and prescription.

According to Jamnick et al. (2020), basing the prescription on fixed percentages of maximal anchors, such as maximal oxygen uptake (VO_2_max), maximum heart rate (HR_max_), or maximum work rate (i.e., maximum power output or velocity), is not recommended as there is a large variability in the physiological responses. However, although there is evidence supporting the validity of submaximal anchors, such as ventilatory thresholds, considered as a gold standard (Gaskill et al., 2001; Keir et al., 2022), or lactate thresholds (Faude et al., 2009), inaccuracies appear to establish them, what could lead to an erroneous training load prescription and distribution within the training domains (Pallarés et al., 2016). Furthermore, these methods can be invasive and expensive and require special equipment and qualified operators, what makes it difficult to have a continuous evaluation during training or repeated evaluations during the training program.

Considering these issues, searching for an alternative to determining exercise intensity thresholds is justifiable. Threshold detection using heart rate (HR) variability (HRV) has been extensively investigated over the past years since HR monitoring presents a relatively simple, non-invasive, and cost-effective option available to the general population (Kaufmann et al., 2023). HRV offers insights into the fluctuations in heart rate from beat to beat and can reveal physiological adaptations in several conditions, including exercise (Manresa-Rocamora et al., 2021). Moreover, apart from its application in determining intensity domains during incremental tests, HRV can be employed in other contexts due to its sensitivity to homeostatic perturbations such as fatigue or psychological factors (Blasco-Lafarga et al., 2017). In fact, when combined with its ease of periodic use throughout a training program, this capability allows for the adjustment of training intensities on a day-to-day basis based on the athlete’s status, as suggested by previous studies (Javaloyes et al., 2019; Schaffarczyk et al., 2022a; Van Hooren et al., 2023).

Validity and reliability of various HRV indexes have been studied for this purpose, such as frequency-domain (Anosov et al., 2000; Cottin et al., 2006; Cottin et al., 2007; Cassirame et al., 2015), time-domain (Karapetian et al., 2008; Candido et al., 2015), and nonlinear indexes (Garcia-Tabar et al., 2013; Candido et al., 2015). While certain indexes demonstrate encouraging outcomes (Karapetian et al., 2008; Garcia-Tabar et al., 2013; Ramos-Campo et al., 2017), drawing conclusions becomes complicated due to various factors: the applicability depends on the specific exercise modality (Cottin et al., 2007), difficulties in identifying thresholds (Cassirame et al., 2015), methodology limitations (i.e., determining the threshold by finding a nadir poses challenges as not all the subjects exhibit easily detectable nadirs) (Blasco-Lafarga et al., 2017), or the loss of dynamic range after the aerobic threshold (Tulppo et al., 1998).

Recently, a nonlinear index of HRV, the short-term scaling exponent alpha 1 of detrended fluctuation analysis (DFA a1) has been proposed as a biomarker for exercise intensity distribution (Rogers et al., 2021a). This dimensionless index is based on HR time series correlation properties, fluctuating between approximately 1.5 and 0.5 values during resting conditions and exercise. A DFA a1 value above 1 represents a non-stationary strongly correlated behaviour, associated with low intensity exercise, recovery, or illness if it appears chronically in rest conditions. On the opposite, a value of 0.5 represents a random and not correlated behaviour, showing small continuous fluctuations without a pattern. Values below 0.5 exhibit an anti-correlated behaviour, getting smaller fluctuations in larger time windows, associated with high-intensity exercise, cardiac risk, or pathologies when it is a chronic status. Values between 0.5 and 1.0 show a positively correlated signal, balancing complete predictability and randomness (Goldberger et al., 2002; Hardstone et al., 2012).

During exercise, DFA a1 has been shown to decrease as the work rate intensifies. Initially, a stable area above values of 1.0 is identified at low intensities, followed by a decline from values of 1.0 to 0.5, and finally flattening at values of 0.5 or below at high intensities (Gronwald et al., 2019; Rogers et al., 2021a). In previous studies, absolute values of 0.75 and 0.5 of DFA a1 (HRTV1 and HRVT2, respectively) were positively associated with first (VT1) and second ventilatory thresholds (VT2), respectively, in a group of male recreational runners during an incremental treadmill test (Rogers et al., 2021a; Rogers et al., 2021c). The association between HRVT1 and VT1 has also been tested in a cardiac disease population performing an incremental cycling ramp test, finding strong correlations (Rogers et al., 2021f). In another population, elite triathletes, HRVT1 closely agreed with the first lactate threshold in an incremental cycling stage protocol (Rogers et al., 2022). Similar outcomes were observed in a group of elite cyclists, revealing significant positive correlations between HRVT1 and LT1, as well as between HRVT2 and LT2 (Mateo-March et al., 2023). Furthermore, both DFA a1 thresholds were assessed in women of any fitness level, showing good agreement with traditional methods (Schaffarczyk et al., 2022b). These results suggested that the utilization of DFA a1 could provide guidance for training intensity distribution, establishing valid domain boundaries without needing gas exchange or blood lactate testing and avoiding exhaustive maximal tests.

However, there is a notable absence of studies assessing the reliability of the DFA a1 method in establishing intensity thresholds. Additionally, existing research has created a gap in our understanding, particularly concerning untrained populations without pathologies. For instance, Schaffarczyk et al. (2022b) included women who either did or did not regularly practice exercise, but the method has not been applied to untrained men. Furthermore, the choice of a test protocol emerges as a crucial factor in determining intensity thresholds, given that outcomes may differ based on the characteristics of the test (Jamnick et al., 2018; Jamnick et al., 2020). Previous investigations utilized protocols such as the Bruce protocol, an incremental treadmill test (Rogers et al., 2021a; Rogers et al., 2021c), or incremental cycling ramp tests (Rogers et al., 2021f; Schaffarczyk et al., 2022b). However, there is a lack of studies employing incremental cycling stage tests for comparing HRV thresholds and ventilatory thresholds. The studies that reported the comparison between HRV thresholds and lactate thresholds (Rogers et al., 2022; Mateo-March et al., 2023), are the only ones that have used incremental cycling stage tests. Although Rogers et al. (2022) conducted a 3-min stage duration incremental cycling test, their evaluation focused solely on the first lactate threshold. Meanwhile, Mateo-March et al. (2023) assessed both first and second lactate thresholds, but the 1-min increments in the incremental cycling test employed are not recommended for accurately identifying lactate thresholds (Pettitt et al., 2013; Jamnick et al., 2018).

Therefore, this study aims to assess the reliability of DFA a1 to determine intensity thresholds in untrained healthy adults during an incremental cycling stage test and their agreement with ventilatory and lactate thresholds. If DFA a1 is reliable and valid to identify intensity thresholds, it could provide an easy, non-invasive method for prescribing and monitoring exercise intensity in untrained populations.

## 2 Materials and methods

### 2.1 Participants

Sixteen healthy volunteers who did not regularly practice any sport or followed a structured physical activity program were recruited for the study. Table 1 presents descriptive data of the participants. All participants were informed about the testing protocols and potential risks, their medical history was reviewed, and then institutionally approved consent was given. Approval for the study was granted by Miguel Hernández University, Spain (CID.DPC.01.21) and conformed to the principles of the Declaration of Helsinki.

**TABLE 1:** Gender, age, body weight, height, and BMI of participants (mean ± *SD*).

	n	Age (years)	BW (kg)	Height (cm)	BMI (kg·m^-2^)
Men	13	24.5 ± 4.2	72.2 ± 8.0	174.0 ± 6.3	23.9 ± 2.6
Women	3	22.0 ± 1.7	58.2 ± 2.7	162.6 ± 10.7	22.1 ± 1.9
Total	16	24.1 ± 3.9	69.6 ± 9.2	171.9 ± 8.2	23.5 ± 2.5

BW: body weight; BMI: body mass index.

### 2.2 Design

The study utilized a repeated measures design, with each participant performing two identical incremental cycling stage tests on a cycle-ergometer (Monark, Exercise AB, Vansbro, Sweden) until volitional exhaustion. The tests were conducted with a 6–9 days interval between them. To minimize the potential influence of confounding factors, participants were instructed to avoid consuming caffeine, alcohol, or any stimulant substances for 4 h prior to testing, and to refrain from performing high intensity exercise the day before the test. Additionally, all testing was conducted at least 2 h post-meal. During both tests, gas exchange kinetics, lactate, and HR were measured.

### 2.3 Incremental cycling stage test

Participants first adjusted the cycle-ergometer setup to their comfortable cycling position by modifying saddle height, handlebar height, saddle to handlebar distance, and pedals. After a 5 min baseline recording of all the variables while sitting in the saddle, the incremental test began with a 3 min warm-up at a workload of 50 W, followed by increases of 15/20/25 W every 3 min until exhaustion, when a 3 min recovery at 50 W was performed. Importantly, the workload increments were individualized with the aim of achieving a standardized duration for the test across participants to improve the validity of lactate thresholds (Jamnick et al., 2018). Despite resulting in varying workload increase ratios among participants, a prior study (Fleitas-Paniagua et al., 2023) indicated that this individualization does not appear to impact the identification of HRV thresholds in HR and VO_2_rel. This individualization was based on the estimation of cycle-ergometer VO_2_max, following the method outlined by Neder et al. (1999) and the mechanical efficiency reported by Hansen et al. (1987) for sedentary people. During the tests, participants maintained a self-selected cadence between 60 and 90 rpm (Gronwald et al., 2018). The test finished when a participant voluntarily stopped or was unable to maintain a constant cadence (dropped more than 10 rpm below their preferred cadence). Strong verbal encouragement was provided during the test to ensure that participants reached their maximal capacity. Blood lactate was sampled at each stage, and gas exchange kinetics and HR were continuously recorded.

Note that for the second test, the cycling position was the same as in the first one. Furthermore, participants were indicated to maintain the same cadence as in the first test.

### 2.4 Gas exchange and ventilatory thresholds determination

**FIGURE 1:**
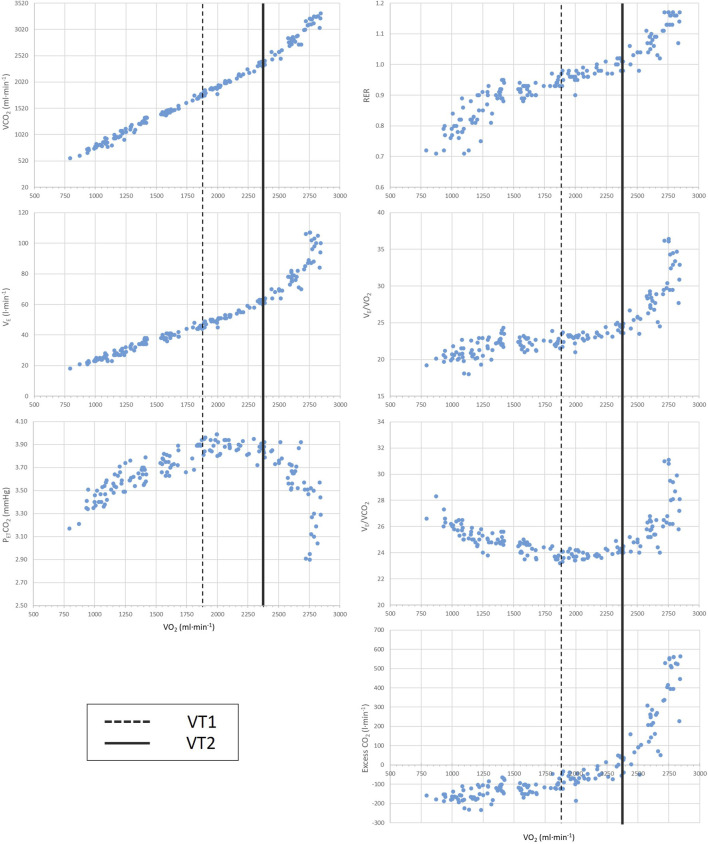
Representative illustration of methods used to determine VT1 and VT2 from an incremental exercise test. VT1: first ventilatory threshold; VT2: second ventilatory threshold; RER: respiratory exchange rate; V_E_: ventilation; VO_2_: oxygen uptake; VCO_2_: carbon dioxide output; P_ET_CO_2_: end tidal carbon dioxide expiration.

**FIGURE 2:**
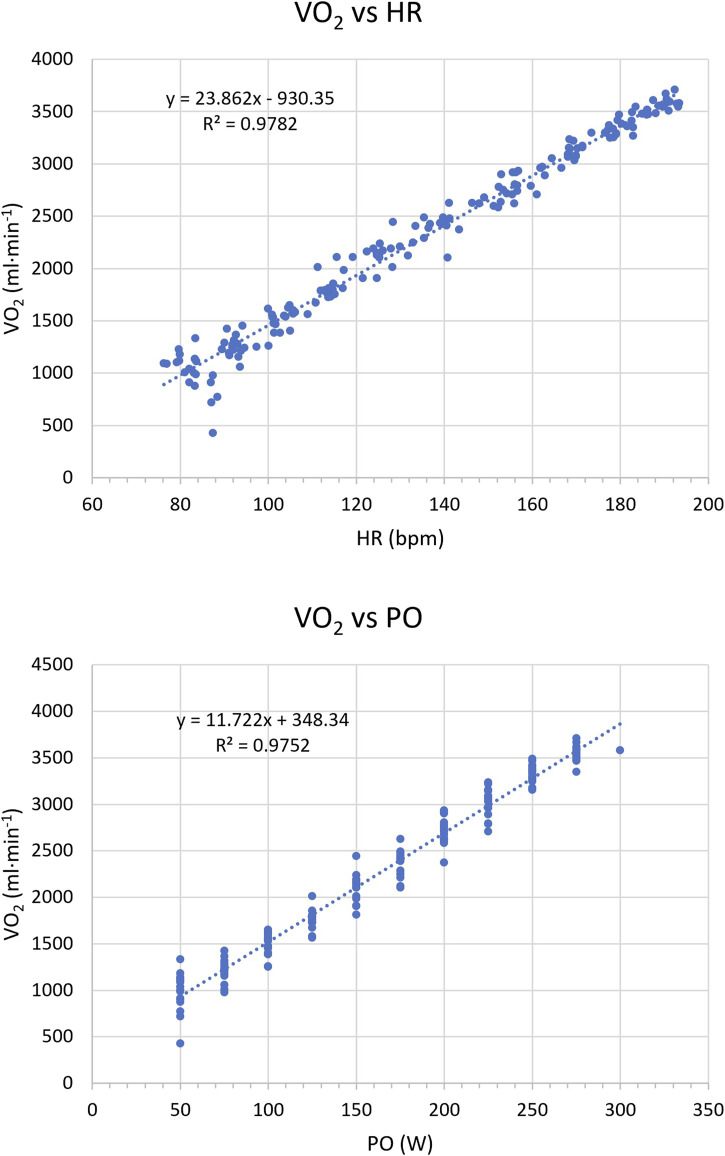
Representative illustration of (top) the VO_2_ vs. HR regression, and (bottom) the VO_2_ vs. PO regression from an incremental exercise test. VO_2_, oxygen uptake; HR, heart rate; PO, power output.

Respiratory gas exchange was measured using MasterScreen CPX (Jaeger, Hoechberg, Germany) on a breath-by-breath basis and calibrated before each testing day. The highest 10-s oxygen uptake (VO_2_) average was used to calculate VO_2_max. Ventilatory thresholds (i.e., VT1 and VT2) were determined using a mixed method to maximize the reliability of the determinations as Keir et al. (2022) suggested. We averaged ventilatory data every 10 s and plotted (Figure 1). Two experienced researchers (NS and JMS) individually checked the VT1 and VT2 values, and any discrepancies were resolved by consensus. HR and power output (PO) at VT1 and VT2 were also calculated for both tests using the VO_2_ vs HR and VO_2_ vs PO linear regression, respectively (Figure 2).

### 2.5 Blood lactate measurement and determination of lactate thresholds

Blood lactate concentration ([La^−^]) was measured using a portable analyser (Lactate Scout, SensLab GmbH, Leipzig, Germany) from the earlobe (Tanner et al., 2010) [La^−^] was measured before the beginning, during the last 30 s of every stage, and at the end of the test. The determination of lactate thresholds was done with ExPhysLab (https://www.exphyslab.com/), a web application which functionality is supported by Lactater R package (Mattioni Maturana, 2023). The first lactate threshold was determined using fixed blood lactate accumulations of 2.0 and 2.5 mmol·L^-1^ (LA 2.0 and LA 2.5, respectively), and the baseline plus method at which lactate increases to 1.0 above baseline values (Bsln+1.0). The second lactate threshold was determined by the onset of blood lactate accumulation of 4.0 mmol l^-1^ (OBLA), and the baseline plus method at which lactate increases to 1.5 mmol l^-1^ above baseline values (Bsln+1.5). We selected these threshold determination methods because they are commonly used in both practical settings and previous research literature, and they have demonstrated validity in delineating intensity domains (Seiler, 2010; Pallarés et al., 2016; Jamnick et al., 2020).

### 2.6 RR measurements and calculation of heart rate variability thresholds

The study used a 3-lead ECG (MP35; Biopac Systems Ltd., California, USA) to continuously monitor HR with a sampling rate of 1,000 Hz. The ECG electrodes were placed in the CM5 distribution after skin cleansing and shaving. Biopac filter settings were set to 0.05 Hz high-pass filter and 150 Hz low-pass filter. Sample data from the MP35 (.acq files) was saved and imported to Kubios HRV Scientific 4.0.1 (Biosignal Analysis and Medical Imaging Group, Department of Physics, University of Kuopio, Kuopio, Finland). Kubios HRV preprocessing settings were at the default values including the RR detrending method which was kept at “Smoothn priors” (Lambda = 500) (Tarvainen et al., 2014). The automatic beat correction filter was applied, and no participant exhibited artifact levels exceeding 5% (see Supplementary Table S3). Consequently, all of them were included in the analysis. To estimate DFA a1, the root mean square fluctuation of the integrated and detrended data was measured in observation windows of different sizes, and the data were plotted against the size of the window on a log-log scale. The scaling exponent represents the slope of the line, which relates (log) fluctuation to (log) window size (Mendonca et al., 2010). DFA a1 window width was set to 4 ≤ *n* ≤ 16 beats.

To calculate DFA a1 thresholds, a value of 0.75 and 0.5 was chosen for HRVT1 and HRVT2, respectively (Rogers et al., 2021a; 2021c). DFA a1 was calculated from the incremental exercise test RR series using 2 min windows with a recalculation every 5 s throughout the test.

**FIGURE 3:**
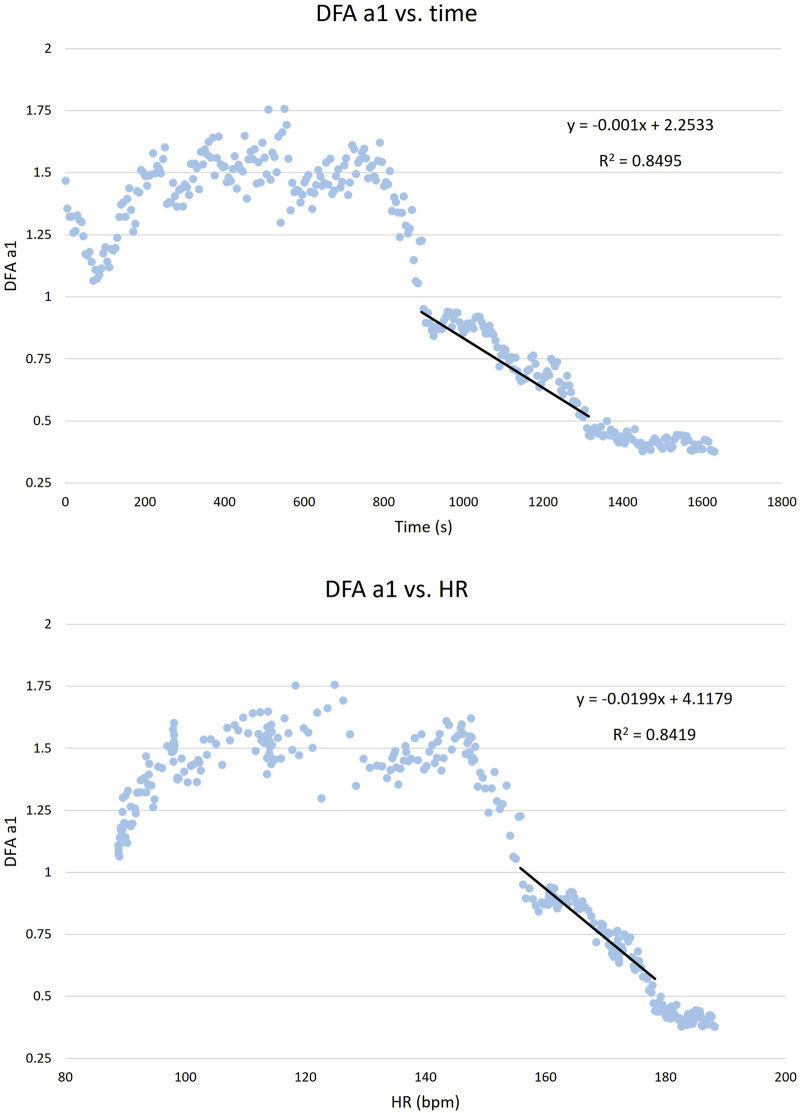
(Top) DFA a1 vs heart rate (HR) and (bottom) DFA a1 vs time. Calculation of the linear regression for values from approximately 1.0 to 0.5. This regression analysis is used to identify the HR and times at which DFA a1 reaches 0.75 and 0.5 values.

To determine HR, relative VO_2_ (VO_2_rel), and PO at HRV thresholds, the method described previously in the literature was followed (Rogers et al., 2021a). For HR, DFA a1 was plotted against HR, and a linear regression was computed for values ranging from approximately 1.0 to 0.5 (Figure 3 (top)). For VO_2_rel and PO, the same method was applied plotting DFA a1 against time (Figure 3 (bottom)) The HR or times at which DFA a1 reached 0.75 and 0.5 were identified using the linear regression equation. Subsequently, the time values were converted to VO_2_rel and PO using the VO_2_ vs time and PO vs time linear regression.

### 2.7 Statistical analysis

Standard statistical methods were used for the calculation of means and standard deviations (*SD*). The normal distribution of data was checked by Shapiro-Wilk’s test. VT1 VO_2_rel did not follow a normal distribution. Parametric and non-parametric comparisons between sessions and methods were conducted using the paired Student’s t-test and the paired Wilcoxon signed-rank test, respectively. To assess the relationship between methods we calculated Pearson’s *r* correlation coefficient for parametric analysis, Spearman’s rho correlation coefficient for non-parametric analysis, intraclass correlation coefficient (ICC), and Bland-Altman analysis. Post-hoc power calculations were performed with the G*power 3.1 (Faul et al., 2007). For the reliability analysis, the ICC, change in the mean, and typical error were used (Hopkins, 2000). The size of Pearson’s *r* correlations was evaluated as follows; *r* < .1 trivial, .1 ≤ *r* < .29 small; .3 ≤ *r* < .49 moderate; .5 ≤ *r* < .69 high; .7 ≤ *r* < .89 very high; .9 ≤ *r* < 1 nearly perfect; and *r* = 1 perfect (Cohen, 2013). ICC was evaluated as; values <.5 poor reliability; values between .5 and .75 moderate reliability; values between .75 and .9 good reliability, and values >.9 excellent reliability (Portney and Watkins, 2009). For all tests, the statistical significance was accepted as *p* ≤ .05. Analysis was performed using JASP v0.18.1.0 (JASP Team, 2019; jasp-stats.org) and Microsoft^®^ Excel 365 for Windows.

## 3 Results

### 3.1 Data inclusion

To compare the methods, we used the data from session 2, using the first session as a familiarization with the set. We performed agreement and reliability analyses with 15 participants as one participant did not perform session 2.

### 3.2 Comparison between sessions

The rest values, maximal values, and HR, VO_2_rel, and PO at thresholds determined by the different methods for session 1 and session 2 are presented in Table 2. We found significant differences between sessions for HRVT1 HR (*p =* .018), HRVT2 HR (*p =* <.001), HRVT2 VO_2_rel (*p* = .008), and HR_max_ (*p* = .01). The individual results of each participant are shown in (Supplementary Tables S1, S2, S3).

**TABLE 2:** Descriptive data and comparison of rest values, maximal values, and HR (bpm), relative VO_2_ (mL·kg^-1^·min^-1^), and PO (W) values at thresholds determined by HRV, gas exchange, and blood lactate methods as mean ± SD.

	Session 1			Session 2		
Rest values						
VO_2_rest	4.9 ± 0.8			5.0 ± 0.4		
HR_rest_	71 ± 11			70 ± 13		
DFA a1_rest_	0.99 ± 0.32			0.96 ± 0.31		
Heart rate variability thresholds						
	**HR**	**VO**_**2**_ **rel**	**PO**	**HR**	**VO**_**2**_ **rel**	**PO**
HRVT1	166.8 ± 10.8	36.0 ± 6.3	166.6 ± 43.2	157.7 ± 13.9^a^	33.8 ± 8.2	159.8 ± 53.9
HRVT2	176.3 ± 9.6	39.9 ± 7.1	188.5 ± 47.9	169.9 ± 10.3^a^	38.4 ± 8.3^a^	185.9 ± 55.4
Ventilatory thresholds						
	**HR**	**VO**_**2**_ **rel**	**PO**	**HR**	**VO**_**2**_ **rel** ^b^	**PO**
VT1^b^	133.5 ± 13.8	27.0 ± 5.3	114.8 ± 33.1	129.1 ± 16.3	26.4 ± 7.5	114.4 ± 40.0
VT2	166.3 ± 13.7	38.2 ± 7.6	178.8 ± 46.4	162.7 ± 14.3	37.5 ± 8.3	180.7 ± 51.2
Lactate thresholds						
	**HR**	**VO**_**2**_ **rel**	**PO**	**HR**	**VO**_**2**_ **rel**	**PO**
LA 2.0	144.4 ± 14.3	31.2 ± 7.5	135.4 ± 46.6	142.4 ± 16.3	31.8 ± 9.0	145.7 ± 50.7
LA 2.5	150.9 ± 13.5	33.3 ± 7.2	147.2 ± 45.7	149.1 ± 15.3	33.1 ± 8.6	149.9 ± 54.9
OBLA	163.8 ± 12.5	37.6 ± 6.9	170.7 ± 44.7	161.0 ± 14.1	37.1 ± 8.4	172.8 ± 55.8
Bsln+1.0	143.8 ± 14.4	31.0 ± 8.0	134.1 ± 47.9	142.6 ± 16.0	31.9 ± 8.2	145.8 ± 46.8
Bsln+1.5	149.9 ± 13.5	33.1 ± 7.7	145.8 ± 46.9	149.1 ± 15.0	33.2 ± 8.1	149.8 ± 52.5
Maximal values						
VO_2_max	49.7 ± 8.0			49.6 ± 7.6		
HR_max_	192 ± 10.0			189 ± 10.0^a^		
PO_max_	229.1 ± 50.4			232.2 ± 53.0		

^b^ Analysed with paired Wilcoxon *t*-test due to its non-normal distribution.

^a^ *p* < .05 between sessions.

HR; heart rate; VO_2_rel, relative oxygen uptake; PO, power output; VO_2_rest, resting oxygen uptake; HR_rest_, resting heart rate; DFA a1_rest_, resting DFA a1; VO_2_rel, relative oxygen uptake; HRVT1, first heart rate variability threshold; HRVT2, second heart rate variability threshold; VT1, first ventilatory threshold; VT2, second ventilatory threshold; LA, lactate accumulation at a fixed value; OBLA, onset of blood lactate accumulation at 4 mmol l^-1^; Bsln+, lactate Baseline + fixed lactate value; VO_2_max, maximal oxygen uptake; HR_max_, maximum heart rate; PO_max_, maximal power output.

### 3.3 Reliability

We present the ICC, change in mean, and typical error for all the thresholds in Table 3. Whereas HRVT1 HR showed a moderate ICC (ICC = .52), VO_2_rel (ICC = .81) and PO (ICC = .87) values had a good ICC. Although ICC of HRVT2 HR was good (ICC = .85), it was excellent for VO_2_rel (ICC = .96), and PO values (ICC = .97).

**TABLE 3:** Reliability results for all the thresholds. Change in mean and typical error are expressed in different units: HR (bpm), VO_2_rel (mL·kg^-1^·min^-1^), and PO (W).

	Change in mean			Typical error		ICC		
		Units	%	Units	%			95% CI
**HR**	**HRVT1**	−7.53	−4.7	8.83	5.5	.52	*moderate*	.07–.79
	**HRVT2**	−5.00	−2.9	4.08	2.4	.85	*good*	.63–.94
	**VT1**	−4.13	−3.1	6.84	5.2	.79	*good*	.50–.92
	**VT2**	−2.87	−1.7	5.78	3.5	.83	*good*	.58–.94
	**LA 2.0**	−1.79	−1.2	6.97	4.9	.80	*good*	.51–.92
	**LA 2.5**	−1.87	−1.2	6.18	4.1	.82	*good*	.55–.93
	**OBLA**	−2.53	−1.6	5.33	3.3	.84	*good*	.60–.94
	**Bsln+1.0**	−1.07	−0.7	8.40	5.9	.71	*moderate*	.34–.89
	**Bsln+1.5**	−0.87	−0.6	7.21	4.8	.76	*good*	.43–.91
**VO**_**2**_ **rel**	**HRVT1**	−2.38	−6.8	3.13	8.9	.81	*good*	.54–.93
	**HRVT2**	−1.81	−4.6	1.61	4.1	.96	*excellent*	.88–.98
	**VT1**	−0.75	−2.8	2.49	9.3	.85	*good*	.62–.95
	**VT2**	−0.95	−2.5	1.95	5.1	.94	*excellent*	.83–.98
	**LA 2.0**	−0.26	−0.8	2.54	8.0	.91	*excellent*	.76–.97
	**LA 2.5**	−0.51	−1.5	2.35	7.0	.92	*excellent*	.77–.97
	**OBLA**	−0.88	−2.3	2.51	6.7	.89	*good*	.72–.96
	**Bsln+1.0**	−0.06	−0.2	2.64	8.3	.90	*excellent*	.74–.96
	**Bsln+1.5**	−0.25	−0.8	2.37	7.1	.91	*excellent*	.77–.97
**PO**	**HRVT1**	−7.07	−4.3	17.61	10.8	.87	*good*	.66–.95
	**HRVT2**	−3.26	−1.7	8.45	4.5	.97	*excellent*	.92–.99
	**VT1**	−0.87	−0.8	13.02	11.3	.88	*good*	.69–.96
	**VT2**	1.20	0.7	11.84	6.6	.94	*excellent*	.85–.98
	**LA 2.0**	2.86	2.0	12.66	8.8	.94	*excellent*	.83–.98
	**LA 2.5**	1.27	0.9	11.66	7.8	.95	*excellent*	.86–.98
	**OBLA**	0.78	0.5	11.92	6.9	.95	*excellent*	.85–.98
	**Bsln+1.0**	3.88	2.7	13.25	9.2	.93	*excellent*	.81–.97
	**Bsln+1.5**	2.46	1.7	11.98	8.1	.94	*excellent*	.85–.98

95% CI, ICC, confidence interval; HR; heart rate; VO_2_rel, relative oxygen uptake; PO, power output; VO_2_rel, relative oxygen uptake; HRVT1, first heart rate variability threshold; HRVT2, second heart rate variability threshold; VT1, first ventilatory threshold; VT2, second ventilatory threshold; LA, lactate accumulation at a fixed value; OBLA, onset of blood lactate accumulation at 4 mmol l^-1^; Bsln+, lactate Baseline + fixed lactate value.

### 3.4 Comparison between methods

**FIGURE 4:**
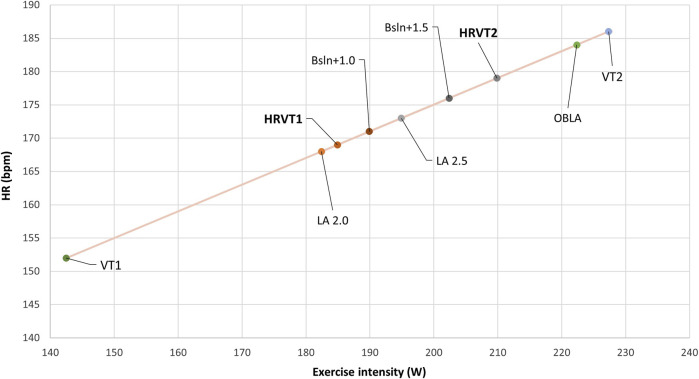
Example of thresholds of one participant determined in HR by HRV, blood lactate, and gas exchange methods. HR, heart rate; HRVT1, first heart rate variability threshold; HRVT2, second heart rate variability threshold; VT1, first ventilatory threshold; VT2, second ventilatory threshold; LA, lactate accumulation at a fixed value; OBLA, onset of blood lactate accumulation at 4 mmol l^-1^; Bsln+, lactate Baseline + fixed lactate value.

Because VT1 VO_2_rel did not follow a normal distribution, the paired Wilcoxon signed-rank test was employed to compare methods, and Spearman’s rho correlation coefficient was used to assess the relationship between VT1 and the other methods. An example of the thresholds determined by each method for one participant is shown in Figure 4.

#### 3.4.1 HRVT1 comparison with ventilatory and lactate thresholds

HRVT1 comparisons, correlations, ICC, and Bland-Altman values are presented in Table 4.

**TABLE 4:** Agreement results. Comparison of HR (bpm), relative VO_2_ (mL·kg^-1^·min^-1^), and PO (W) at HRVT1 with the different used methods.

		HRVT1								
		Mean differences		*r* Pearson/*rho* spearman			ICC	95% CI	Bland Altman	
		*p*-value	Power	*r/rho* value	*p*-value	Power			Bias (bpm)	SD (bpm)
**HR**	**VT1**	<.001	1.000	.314	.254	.352	.310	−.19–.68	28.3	17.4
	**LA 2.0**	<.001	.988	.695	.006	.901	.677	.31–.87	15.0	12.3
	**LA 2.5**	.011	.742	.697	.004	.922	.693	.33–.88	8.4	11.6
	**Bsln+1.0**	<.001	.995	.735	.003	.948	.718	.38–.89	14.7	11.1
**VO**_**2**_ **rel**	**VT1^a^**	<.001	.996	.696	.005	.871	.753	.43–.91	7.3	5.5
	**LA 2.0**	.026	.639	.887	<.001	.997	.885	.70–.96	4.1	6.5
	**LA 2.5**	.546	.089	.891	<.001	.999	.894	.72–.96	0.6	4
	**Bsln+1.0**	.025	.651	.882	<.001	.997	.887	.71–.96	4	6.4
**PO**	**VT1**	<.001	.997	.762	<.001	.948	.730	.38–.90	45.4	34.9
	**LA 2.0**	.003	.915	.899	<.001	.999	.916	.78–.97	23.8	23.4
	**LA 2.5**	.090	.396	.925	<.001	1.000	.925	.80–.97	9.9	21.1
	**Bsln+1.0**	.003	.920	.892	<.001	.998	.913	.77–.97	23.7	23.1

^a^ Analysed with paired Wilcoxon *t*-test and Spearman’s rho correlation coefficient due to its non-normal distribution.

95% CI, ICC, confidence interval; HR; heart rate; VO_2_rel, relative oxygen uptake; PO, power output; VO_2_rel, relative oxygen uptake; HRVT1, first heart rate variability threshold; VT1, first ventilatory threshold; LA, lactate accumulation at a fixed value; OBLA, onset of blood lactate accumulation at 4 mmol l^-1^; Bsln+1.0, lactate baseline +1.0 mmol·L^-1^.

HRVT1 exhibited a significant difference compared to all the methods, except for LA 2.5 in VO_2_rel (*p* = .546) and PO (*p* = 0.090) values. Notably, LA 2.5 PO showed a nearly perfect correlation (*r* = .925, *p* < .01), and LA 2.5 VO_2_rel correlation was very high (*r* = .891, *p* < .01). Despite the significant differences, LA 2.5 HR demonstrated a high correlation (*r* = .697, *p* < .01), although not surpassing but similar to Bsln+1.0 (*r* = .735, *p* < .01). Regarding LA 2.5, the ICC was moderate for HR (ICC = .693), good for VO_2_rel (ICC = .894), and excellent for PO (ICC = .925). Once again, in HR results, Bsln+1.0 showed a higher but comparable ICC (ICC = .718). Moreover, when comparing HRVT1 with the LA 2.5 method, the lowest bias was observed, with values of 8.4 ± 11.6 bpm, 0.6 ± 4.0 mL·kg^-1^·min^-1^, and 9.9 ± 21.1 W.

#### 3.4.2 HRVT2 comparison with ventilatory and lactate thresholds

HRVT2 comparisons, correlations, ICC, and Bland-Altman values are presented in Table 5.

**TABLE 5:** Agreement results. Comparison of HR (bpm), relative VO_2_ (mL·kg^-1^·min^-1^), and PO (W) at HRVT2 with the different used methods.

		HRVT2								
		Mean differences		*r* Pearson			ICC	95% CI	Bland Altman	
		*p*-value	Power	*r* value	*p*-value	Power			Bias (bpm)	SD (bpm)
**HR**	**VT2**	.017	.755	.692	.004	.935	.656	.27–.86	7.5	10.1
	**OBLA**	.006	.891	.665	.007	.913	.632	.23–.85	9.2	10.4
	**Bsln+1.5**	<.001	1.000	.681	.005	.929	.634	.24–.85	21.1	10.8
**VO**_**2**_ **rel**	**VT2**	.450	.112	.881	<.001	.998	.884	.70–.96	0.8	4.1
	**OBLA**	.226	.219	.888	<.001	.999	.888	.71–.96	1.3	4.0
	**Bsln+1.5**	<.001	.993	.872	<.001	.997	.872	.67–.95	5.1	4.2
**PO**	**VT2**	.367	.140	.919	<.001	1.000	.917	.78–.97	5.3	21.9
	**OBLA**	.026	.637	.932	<.001	1.000	.932	.82–.98	13.1	20.5
	**Bsln+1.5**	<.001	1.000	.882	<.001	1.000	.917	.78–.97	49.9	21.7

95% CI, ICC, confidence interval; HR; heart rate; VO_2_rel, relative oxygen uptake; PO, power output; VO_2_rel, relative oxygen uptake; HRVT2, second heart rate variability threshold; VT2, second ventilatory threshold; OBLA, onset of blood lactate accumulation at 4 mmol l^-1^; Bsln+1.5, lactate baseline +1.5 mmol·L^-1^.

HRVT2 HR demonstrated significant differences when compared to all the methods. In contrast, HRVT2 VO_2_rel (*p* = .450) and HRVT2 PO (*p* = .367) exhibited no differences with VT2 VO_2_rel and VT2 PO. Additionally, OBLA VO_2_rel did not show significant differences with HRVT2 VO_2_rel. Despite the significant differences in HR values, HRVT2 displayed strong correlations with VT2, characterized as high in HR (*r* = .692, *p* < .01), very high in VO_2_rel (*r* = .881, *p* < .01), and nearly perfect in PO (*r* = .919, *p* < .01). The ICC was moderate for HR (ICC = .656), good for VO_2_rel (ICC = .884), and excellent for PO (ICC = .917). Furthermore, the comparison between HRVT2 and the VT2 method revealed the lowest bias, with values of 7.5 ± 10.1 bpm, 0.8 ± 4.1 mL kg^-1^·min^-1^, and 5.3 ± 21.9 W. Concerning lactate methods, the best correlation and ICC for HR were observed with Bsln+1.5 (*r* = .681, *p* < .01, ICC = .634). OBLA was identified as the method with the highest correlation and ICC with HRVT2 in VO_2_rel (*r* = .888, *p* < .01, ICC = .888) and PO (*r* = .932, *p* < .01, ICC = .932).

## 4 Discussion

The aim of this study was to evaluate the validity and reliability of HRV thresholds in untrained healthy adults using DFA a1 fixed values (0.75 and 0.5) proposed by Rogers et al., 2021a; Rogers et al., 2021c). Our results indicate that HRV thresholds exhibit good reliability, comparable to or surpassing some methods examined. Notably, PO reliability yielded the best outcomes across all methods. In terms of agreement, HRV thresholds demonstrated high concordance with ventilatory and lactate methods for HR, VO_2_rel, and PO, with the strongest agreement observed in PO values and the weakest in HR values, the latter showing even significant differences with all the compared methods. Specifically, HRVT1 showed the highest agreement with LA 2.5 in W, while HRVT2 exhibited optimal agreement with VT2 in W. These findings underscore the validity and reliability of HRV thresholds, offering practical implications for exercise testing and prescription in clinical and athletic contexts.

### 4.1 Reliability of the methods

Firstly, it is noteworthy that there were significant differences in the mean comparison between sessions 1 and 2 for both HRVT1 and HRVT2. While the reliability analysis revealed good ICCs for HRVT1 across VO_2_rel and PO, but moderate for HR. In the case of HRVT2, the ICCs were excellent for VO_2_rel and PO but good for HR. Similarly, the reliability outcomes observed in ventilatory, and lactate thresholds demonstrated ICCs ranging from moderate to excellent. Notably, HRVT2 exhibited the highest ICCs in VO_2_rel, and PO compared to all the methods considered in this study. The lower ICC values for HR could be attributed to its day-to-day variability, consistent with findings in previous studies, where it has been shown better reliability results for external load parameters (i.e., PO and velocity), as well as VO_2_rel, in comparison to HR (Pfitzinger and Freedson, 1998). Nevertheless, these reliability levels are generally considered sufficiently high for the reliable application of these methods. Other studies examining the reliability of traditional thresholds have reported ICC values ranging from .57 to .96 for different ventilatory and lactate methods applied to determine thresholds in PO values (Pallarés et al., 2016). Our results align with this range, spanning from .52 to .97.

When analysing the change in mean, HRVT1 displayed the highest values across all measures: HR, VO_2_rel, and PO (−4.7%, −6.8%, and −4.3%, respectively). In contrast, HRVT2 showed values similar to other methods in HR (−2.9%) and PO (−1.7%), with a slight increase for VO_2_rel (−4.6%). However, these variations fall within acceptable practical ranges. Regarding typical error, a consistent pattern emerged: HRVT2 consistently demonstrated the lowest typical error for all measures (HR = 2.4%, VO_2_rel = 4.1%, PO = 4.5%), whereas HRVT1 exhibited values comparable to those of other methods.

We have considered the recommendation to use a combination of methods for determining ventilatory thresholds to enhance reliability (Jamnick et al., 2020; Keir et al., 2021). Additionally, when the population is untrained and lacks experience with a cycle-ergometer, as is the case in this study, conducting familiarization trials is recommended to further improve reliability (Muniz-Pumares et al., 2019). We acknowledge that had we considered this factor, the outcomes might have been even more favourable.

Considering all results, PO emerges as a preferable choice due to its practicality for field use in prescribing and monitoring training program intensity.

### 4.2 HRVT1 agreement with ventilatory and lactate thresholds

The existing literature presents inconsistencies and heterogeneity regarding the agreement between HRVT1 and VT1. In a study involving women during an incremental cycling protocol, significant differences between VT1 and HRVT1 were reported (Schaffarczyk et al., 2022b). In contrast, in different studies, Rogers et al. (2021a; Rogers et al., 2021f) did not find significant differences between these methods. Despite the significant differences reported by Schaffarczyk et al. (2022b), there was a very high correlation and good ICC between methods measured with HR (*r* = .87, ICC = .87) and VO_2_rel (*r* = .81, ICC = .77). Rogers et al. (2021a) similarly reported good agreement between HRVT1 and VT1 in HR (*r* = .97, ICC = .96) and VO_2_rel (*r* = .99, ICC = .99) during an incremental treadmill test with male recreational runners. When the DFA a1 method was applied to patients with cardiovascular disease during an incremental cycling ramp test, strong correlations between VT1 and HRVT1 were observed in VO_2_rel (*r* = .95), PO (*r* = .87), and HR (*r* = .86) (Rogers et al., 2021f).

Contrastingly, the results of our study revealed a lack of agreement between HRVT1 HR and VT1 HR, evidenced by significant differences (*p* < .01), a moderate correlation (*r* = .31), a poor ICC (ICC = .31), and a substantial bias of 28.3 (±17.4) bpm. Despite significant differences, when thresholds were measured with VO_2_rel and PO, the correlations were high and very high (*r* = .70, *r* = .76), and the ICC was good and moderate (ICC = .75, ICC = .73), respectively, although bias remained considerable (7.3 ± 5.5 mL kg^-1^·min^-1^, 45.4 ± 34.9 W). This aligns with Rogers et al. (2021f), who demonstrated stronger correlations in VO_2_rel and PO. However, it is noteworthy that their HR results showed higher values and greater similarity between methods, and their bias were comparatively smaller (3.4 ± 7.3 bpm, 1.20 ± 2.9 mL kg^-1^·min^-1^, and 5.4 ± 12.8 W), as observed in the studies of Rogers et al. (2021a) (−1.9 ± 5.3 bpm, and −0.33 ± 1.3 mL kg^-1^·min^-1^), and Schaffarczyk et al. (2022b) (−4.7 ± 2.3 bpm, −1.3 ± 2.4 mL kg^-1^·min^-1^).

It is worth noting that, even though not all prior studies indicate significant differences between HRVT1 and VT1, there is a consistent trend of HRVT1 reporting slightly higher values compared to VT1 across these studies. A plausible physiological hypothesis for the delayed appearance of DFA a1 thresholds compared to ventilatory thresholds may be linked to the intricate interplay among respiratory, cardiovascular, and neuromuscular control during exercise. Autonomic adjustments to exercise involve several neural mechanisms working together to precisely regulate cardiovascular changes in an intensity-dependent manner. Neural signals originating from chemoreceptors and stretch receptors in the carotid and aortic bodies, metabolically sensitive afferents from skeletal muscle, mechanically sensitive stretch receptors in the cardiopulmonary region, and metabolically sensitive afferents from respiratory muscles are processed within brain cardiovascular control areas, influencing efferent sympathetic and parasympathetic nerve activity (Fisher et al., 2015). These autonomic adjustments, reflected in the structure of HRV and the changes in DFA a1 (Gronwald et al., 2020), elicit alterations in cardiac function during exercise. Ventilatory thresholds, primarily reflective of respiratory responses, may manifest earlier due to the immediate demand for increased oxygen uptake and closer alignment with the demands for carbon dioxide clearance (Ward, 2007). Conversely, DFA a1 thresholds, associated with the autonomic control of the heart, might emerge later as a result of central regulation of heart rate. This delayed onset suggests a subtle integration of cardiac adjustments to maintain stability and efficiency as exercise intensity progresses. Further exploration of the temporal relationship between these thresholds could provide valuable insights into the intricate coordination of physiological systems during exercise.

Regarding lactate thresholds, previous literature has evaluated the agreement between lactate and DFA a1 derived first thresholds. In a study involving elite triathletes during an incremental ramp cycling test, Rogers et al. (2022) used this method, revealing no disparities between the methods. They reported a high correlation between LT1 HR and HRVT1 HR (*r* = .77) and nearly perfect correlation between LT1 power and HRVT1 power (*r* = .98). Additionally, the bias was minimal (−1.7 ± 7.7 bpm and −5.3 ± 10.4 W). In a similar population, elite cyclists, Mateo-March et al. (2023) observed high (*r* = .66) and very high (*r* = .85) correlations, along with moderate (ICC = .64) and good (ICC = .86) ICC values between the first lactate threshold and HRVT1 measured with HR and PO, respectively. Our study showed similar results when comparing HRVT1 and LA 2.5, presenting superior correlations and ICC with PO values (*r* = .93, ICC = .93) than with HR values (*r* = .70, ICC = .69). Better results were also observed when determined with VO_2_rel (*r* = .89, ICC = .89). However, Bland-Altman analysis of LA 2.5 and HRVT1 reported a low but higher bias (8.4 ± 11.6 bpm, 9.9 ± 21.1 W, −0.6 ± 4.0 mL kg^-1^·min^-1^) than in previous studies analysing first lactate and HRV thresholds. Nevertheless, caution is warranted in comparing these findings across studies due to the utilization of different methods to determine lactate thresholds. Rogers et al. (2022) employed the Log-log method, while Mateo-March et al. (2023) did not clearly specify the method, although it appears they used Bsln + methods, likely Bsln+0.5. Furthermore, Mateo-March et al. (2023) used a non-recommended protocol for determining lactate thresholds, employing 1-min stages, whereas the literature suggests at least 3-min stages (Bentley et al., 2007; Jamnick et al., 2020).

Once again, the findings suggest that the use of PO values may be the optimal choice, exhibiting superior correlations and agreement. This observation could potentially be elucidated by the notion that PO, as an external load variable, is less susceptible to the influence of stress factors, dehydration, and other considerations that may impact HR and, to a lesser extent, VO_2_rel, both of which are internal load variables (Fisher et al., 2015).

### 4.3 HRVT2 agreement with ventilatory and lactate thresholds

In contrast to findings in previous literature (Rogers et al., 2021c; Schaffarczyk et al., 2022b), our study revealed significant differences between VT2 HR and HRVT2 HR (*p* < .05). Despite these differences, a high correlation between the two methods was observed (*r* = .69), with a moderate ICC (*r* = .66) and Bland-Altman analysis indicating a low bias of 7.5 (±10.1) bpm. These outcomes are slightly worse than results reported by Rogers et al. (2021c), who identified very high correlations (*r* = .78) and minimal bias (−4 ± 10 bpm) between VT2 HR and HRVT2 HR. Similarly, Schaffarczyk et al. (2022b), demonstrated a comparable low bias (0.5 ± 5.7 bpm) but higher correlation results (*r* = .90) and ICC (ICC = .90). The observed differences in HR results may be attributed to the use of different recording devices, as Rogers et al. (2021c) and Schaffarczyk et al. (2022b) employed a chest strap (Polar H7 and H10, respectively) rather than an ECG. Research has shown that data from a chest strap can exhibit significant differences compared to ECG data in HR measurement during an incremental exercise test. This difference led to lower DFA a1 values during dynamic exercise, particularly in the uncorrelated range, potentially resulting in earlier appearance of HRV thresholds (Rogers et al., 2021d). In alignment with our ECG-recorded results, HRVT2 reported higher values of VT2, suggesting that lower values with the chest strap could contribute to better agreement. Furthermore, Schaffarczyk et al. (2022b) also determined thresholds with VO_2_rel values, reporting very high correlation (*r* = .86), good ICC (ICC = .84), and low bias (−0.4 ± 2.3 mL kg^-1^·min^-1^). These findings align with our results, showing very high correlations (*r* = .88), good ICC (ICC = .88) and low bias (0.8 ± 4.1 mL kg^-1^·min^-1^) between HRVT2 VO_2_rel and VT2 VO_2_rel. Additionally, we provide PO results for HRVT2 and VT2, where we identified the best agreement between methods (*p* = .37, *r* = .92, ICC = .92, bias = 5.3 ± 21.9 W).

In the context of lactate thresholds, only one preceding study has evaluated the agreement between HRVT2 and second lactate threshold (Mateo-March et al., 2023). This study used Bsln+2.0 as second lactate threshold, a value probably around 3 mmol l^-1^. Their findings showed significant differences between methods concerning HR and PO values. Additionally, correlations and ICC exhibited higher values for PO (*r* = .93, ICC = .92) compared to HR values (*r* = .71, ICC = .67). Our study aligns with these results, revealing a lower agreement in HR values (*p* < .01, *r* = .67, ICC = .63) compared to PO values, where we observed no significant differences between methods, and a nearly perfect correlation (*r* = .93) along with an excellent ICC (ICC = .93). Moreover, consistent with the aforementioned comparisons, the correlation between HRVT2 and OBLA in PO values emerged as the strongest (*r* = .93, ICC = .93), although significant differences were identified between methods. The Bland-Altman analysis indicated a lower bias in lactate thresholds for OBLA, though slightly higher than the observed for ventilatory thresholds (9.2 ± 10.4 bpm, 1.3 ± 4.0 mL kg^-1^·min^-1^, and 13.1 ± 20.5 W). While no other study directly compared these thresholds, Gronwald et al. (2019) explored the response of lactate and DFA a1 during an incremental cycling test with trained cyclists. Based on their presented results, it can be inferred that OBLA would occur at a DFA a1 value of approximately 0.46, closely resembling the DFA a1 second threshold of 0.5. The aforementioned results support the good agreement between HRVT2 and OBLA.

As exposed before, the subtle differences in our study when contrasted with previous literature may be due to different reasons. One reason could be the multiple different methods that can be chosen to calculate VTs and LTs, as it is well-documented that depending on the method used, individual results can vary by approximately 30% (Jamnick et al., 2020). Furthermore, in our study, this controversy could be due to the population that participated, as it is the first time this method is applied to untrained healthy male participants with no prior experience with the cycle-ergometer. We tried to avoid the no-experience issue by obtaining the validity results from the second session so that the first one could be considered as a familiarization. Due to the good reliability shown by DFA a1 methods, as well as this index potential to be measured in real-time during training through different applications and to minimize the inconveniences of performing maximal tests, it seems to be a good candidate as a tool or method to determine different intensity domains or training zones.

### 4.4 Limitations, practical applications, and future directions

The use of DFA a1 may introduce certain biases, such as artifact correction (Rogers et al., 2021d), fatigue, stress (Rogers et al., 2021e), and inappropriate detrending (Voss et al., 2015). However, our study has a notable strength in terms of RR interval quality, due to the use of an ECG with a high sample rate and the artifact percentage was very low. To minimize the impact of fatigue on DFA a1 results, participants were specifically instructed to rest adequately (Rogers et al., 2021e). Additionally, we employed Kubios HRV software to ensure appropriate detrending, following the methods outlined by Rogers et al. (2021a).

However, this study has several limitations that warrant acknowledgment. The HRV thresholds were determined using fixed values of 0.75 and 0.5, as proposed in the literature (Rogers et al., 2021a; 2021c; Rogers and Gronwald, 2022). However, these fixed values were chosen for their mathematical significance: 0.5 represents white noise, random behaviour of interbeat pattern and drop below this value representing an anticorrelated range at the very highest work rates, which could be seen as a protective feedback and stabilizing mechanism where interactions and/or coordination of subsystems fail before the whole system fails (Hardstone et al., 2012; Gronwald and Hoos, 2020), and 0.75 is the midpoint between this random behaviour value of 0.5 (seen with high intensity exercise) and a fractal behaviour of the HR time series of 1.0 (seen with very light exercise). It is essential to note that the use of fixed values, while providing a practical approach, introduces a level of simplification that may not fully capture the complexity of HRV dynamics. Further exploration and validation of these thresholds under diverse exercise conditions are necessary to enhance the robustness and generalizability of the findings.

Another limitation of the present study is the reduced group sample size, with only 3 women tested, preventing a definitive conclusion for this population. Nevertheless, it is important to note that statistical power calculations have been conducted for all correlations and method comparisons, yielding high or very high power values. High statistical power enhances the confidence in the study’s findings, suggesting a robust ability to detect true effects or relationships when they exist in the population under study.

Another issue to consider is that we did not calculate the glycogen storage of participants as a dietary recall was not demanded, so this variable was not controlled and could have affected blood lactate measures (Bergström et al., 1967), same as hydration status (Logan-Sprenger et al., 2015).

Despite the observed variations in mean changes, the practical applications of DFA a1 thresholds should not be overlooked. DFA a1 measurement seems more attractive because of its simplicity, cost-effectiveness, and accessibility. It is a non-invasive alternative for the general population, who can use available wearable technology to measure it. This tool has the potential to enable field tests to be carried out to delimit thresholds for performance evaluation and training domain purposes, without the need for a laboratory preset or expensive equipment, and given its dimensionless nature, it should not be needed a calibration with gas exchange. Moreover, actual smartwatches and smartphones can calculate DFA a1 in real-time, enabling practitioners to monitor their training intensity during training, and adjust it in accordance with the session purpose. Looking ahead, the integrating technological advancements for real-time monitoring and feedback could further optimize the practical utility of DFA a1 thresholds in various training scenarios.

For future research, it would be valuable to examine the behaviour of DFA a1 and other physiologic variables during constant load exercise within different intensity domains. This analysis would allow, by means of comparison between DFA a1 and the response of these variables, the analysis of whether their comparability adjusts to the expected in each domain. In addition, it remains unknown whether changes in HRV thresholds after a training period correspond to changes and adaptations in VTs or LTs. The only study evaluating the post-intervention change in HRVT1 vs VT1 showed a reasonable correlation (*r* = 0.72) in a cardiac population (Rogers et al., 2021f), but future research should further investigate this issue. Another area of interest is the application of this method in field tests, whose validity could lead to a great advantage for practitioners and coaches in the regulation and monitoring of intensity training distribution.

## 5 Conclusion

Our findings advocate for the validity and reliability of DFA a1 thresholds in untrained populations, as evidenced by their comparable levels of reliability and agreement with traditional thresholds. Notably, HRVT1 and HRVT2 demonstrated the highest agreement with LA 2.5 and VT2, respectively. Furthermore, the utilization of PO values is recommended for threshold determination. Importantly, DFA a1 derived thresholds offer a distinct advantage over traditional methods, emphasizing their accessibility and cost-effectiveness for real-time monitoring in various training scenarios.

## Data Availability

The original contributions presented in the study are included in the article/Supplementary Material, further inquiries can be directed to the corresponding author.
