# Supplementary material for: Reliability and validity of a non-linear index of heart rate variability to determine intensity thresholds
Source: Front Physiol. 2024 Feb 5;15:1329360. doi: 10.3389/fphys.2024.1329360 (PMC10875128; doi:10.3389/fphys.2024.1329360)
Supplement: Supplementary file 1 [file Table1.docx]

Supplementary Material

**TABLE S1**. Ventilatory thresholds in session 1 and session 2 with measures of HR (bpm), relativeVO_2_ (ml·kg^-1^·min^-1^), and power output (W) for each participant.

| Participant number | VT1 | | | | | | VT2 | | | | | |
| --- | --- | --- | --- | --- | --- | --- | --- | --- | --- | --- | --- | --- |
|  | **S1** | | | **S2** | | | **S1** | | | **S2** | | |
|  | **HR** | **VO_2_rel** | **PO** | **HR** | **VO_2_rel** | **PO** | **HR** | **VO_2_rel** | **PO** | **HR** | **VO_2_rel** | **PO** |
| **1** | 150 | 38.1 | 159.0 | 152 | 42.8 | 179.0 | 193 | 57.2 | 263.0 | 179 | 53.7 | 239.0 |
| **2** | 147 | 22.4 | 58.0 | 155 | 23.4 | 59.0 | 175 | 32.6 | 100.0 | 185 | 33.3 | 108.0 |
| **3** | 127 | 29.3 | 138.0 | 121 | 27.5 | 132.0 | 150 | 39.1 | 196.0 | 147 | 37.3 | 191.0 |
| **4** | 134 | 22.8 | 94.0 | 130 | 22.8 | 99.0 | 155 | 29.9 | 133.0 | 150 | 29.2 | 137.0 |
| **5** | 111 | 19.2 | 64.0 | 109 | 19.9 | 75.0 | 149 | 30.7 | 131.0 | 145 | 30.6 | 139.0 |
| **6** | 151 | 26.8 | 98.0 | 133 | 19.3 | 65.0 | 177 | 33.4 | 125.0 | 166 | 27.7 | 102.0 |
| **7** | 129 | 36.5 | 150.0 | 144 | 41.3 | 182.0 | 165 | 51.9 | 228.0 | 175 | 53.6 | 246.0 |
| **8** | 135 | 26.1 | 123.0 | 115 | 20.9 | 98.0 | 177 | 39.2 | 199.0 | 173 | 38.0 | 202.0 |
| **9** | 120 | 23.7 | 83.0 | 126 | 26.0 | 107.0 | 156 | 38.2 | 158.0 | 151 | 35.9 | 162.0 |
| **10** | 137 | 23.4 | 107.0 | - | - | - | 176 | 34.4 | 169.0 | - | - |  |
| **11** | 119 | 31.3 | 161.0 | 121 | 33.1 | 172.0 | 149 | 43.8 | 239.0 | 155 | 45.6 | 260.0 |
| **12** | 122 | 21.7 | 88.0 | 112 | 17.7 | 76.0 | 166 | 34.0 | 175.0 | 147 | 27.4 | 143.0 |
| **13** | 141 | 25.0 | 100.0 | 134 | 23.7 | 101.0 | 167 | 31.7 | 137.0 | 168 | 33.1 | 157.0 |
| **14** | 115 | 27.1 | 127.0 | 103 | 22.9 | 111.0 | 152 | 41.0 | 215.0 | 154 | 41.3 | 231.0 |
| **15** | 142 | 31.5 | 132.0 | 130 | 29.2 | 118.0 | 167 | 39.8 | 177.0 | 160 | 38.4 | 167.0 |
| **16** | 156 | 26.4 | 154.0 | 152 | 26.2 | 142.0 | 186 | 34.6 | 216.0 | 186 | 37.8 | 226.0 |

S1, session 1; S2, session 2; HR, heart rate; VO_2_rel, relative oxygen uptake; PO, power output; VT1, first ventilatory threshold; VT2, second ventilatory threshold.

**TABLE S2**. Lactate thresholds in session 1 and session 2 with measures of HR (bpm), relative VO_2_ (ml·kg^-1^·min^-1^), and power output (W) for each participant.

|  | LA 2.0 | | | | | | LA 2.5 | | | | | | Bsln+1.0 | | | | | |
| --- | --- | --- | --- | --- | --- | --- | --- | --- | --- | --- | --- | --- | --- | --- | --- | --- | --- | --- |
|  | **S1** | | | **S2** | | | **S1** | | | **S2** | | | **S1** | | | **S2** | | |
|  | **HR** | **VO_2_rel** | **PO** | **HR** | **VO_2_rel** | **PO** | **HR** | **VO_2_rel** | **PO** | **HR** | **VO_2_rel** | **PO** | **HR** | **VO_2_rel** | **PO** | **HR** | **VO_2_rel** | **PO** |
| **1** | 167 | 45.8 | 201.0 | 175 | 52.6 | 229.7 | 172 | 47.8 | 211.8 | 178 | 54.0 | 237.5 | 169 | 46.6 | 205.3 | 173 | 51.8 | 225.1 |
| **2** | 154 | 25.5 | 61.7 | - | - | - | 159 | 27.0 | 68.7 | 162 | 25.7 | 55.8 | 152 | 24.6 | 57.8 | - | - | - |
| **3** | 122 | 28.3 | 126.7 | 118 | 27.1 | 128.2 | 127 | 30.1 | 137.1 | 124 | 29.4 | 140.6 | 127 | 30.1 | 137.1 | 120 | 28.0 | 133.2 |
| **4** | 126 | 20.3 | 76.0 | 121 | 20.2 | 76.8 | 138 | 24.2 | 95.9 | 129 | 22.7 | 91.9 | 122 | 18.8 | 67.8 | 121 | 20.2 | 76.8 |
| **5** | 139 | 28.9 | 108.3 | 143 | 30.3 | 128.3 | 146 | 31.0 | 120.8 | 148 | 31.6 | 136.2 | 134 | 27.7 | 100.8 | 134 | 27.8 | 113.4 |
| **6** | 123 | 19.4 | 63.5 | 126 | 16.9 | 50.2 | 131 | 21.8 | 72.9 | 135 | 19.3 | 60.7 | 120 | 18.5 | 59.7 | 138 | 20.2 | 64.9 |
| **7** | 138 | 40.5 | 164.9 | 139 | 39.8 | 169.3 | 143 | 42.7 | 175.9 | 143 | 41.3 | 177.0 | 141 | 41.9 | 171.8 | 138 | 39.4 | 167.4 |
| **8** | 171 | 37.2 | 183.0 | 148 | 31.4 | 155.8 | 175 | 38.5 | 190.8 | 156 | 33.6 | 169.2 | 166 | 36.0 | 175.2 | 141 | 29.1 | 142.4 |
| **9** | 143 | 32.7 | 123.4 | 132 | 28.9 | 114.4 | 149 | 35.7 | 138.4 | 137 | 31.1 | 126.6 | 143 | 32.7 | 123.4 | 136 | 30.8 | 125.1 |
| **10** | 139 | 24.6 | 105.6 | - | - | - | 151 | 28.2 | 125.1 | - | - | - | 137 | 23.9 | 101.7 | - | - | - |
| **11** | 140 | 40.3 | 210.8 | 148 | 43.0 | 233.7 | 145 | 42.2 | 222.5 | 153 | 44.7 | 245.2 | 139 | 39.5 | 206.1 | 145 | 41.9 | 226.8 |
| **12** | 155 | 31.1 | 153.1 | 148 | 27.5 | 140.9 | 163 | 33.2 | 167.3 | 155 | 29.4 | 153.5 | 154 | 30.7 | 150.2 | 150 | 28.1 | 144.5 |
| **13** | 148 | 27.1 | 108.8 | 153 | 29.3 | 125.4 | 157 | 29.4 | 121.5 | 158 | 30.9 | 134.2 | 153 | 28.5 | 116.4 | 158 | 30.9 | 134.2 |
| **14** | 138 | 35.8 | 179.8 | 133 | 34.4 | 179.2 | 142 | 37.2 | 188.9 | 138 | 36.1 | 190.5 | 138 | 35.5 | 178.0 | 130 | 33.1 | 170.4 |
| **15** | 154 | 35.6 | 152.2 | 141 | 32.7 | 132.9 | 158 | 36.9 | 159.2 | 147 | 34.6 | 142.8 | 156 | 36.1 | 155.0 | 142 | 33.1 | 134.9 |
| **16** | 154 | 26.0 | 147.9 | 168 | 31.3 | 174.6 | 159 | 27.3 | 157.9 | 173 | 33.0 | 186.9 | 150 | 24.9 | 140.0 | 171 | 32.3 | 182.0 |

S1, session 1; S2, session 2; HR, heart rate; VO_2_rel, relative oxygen uptake; PO, power output; LA, lactate accumulation at a fixed value: OBLA, onset of blood lactate accumulation at 4.0 mmol·l^-1^; Bsln+1.0, Baseline +1.0 mmol/l; Bsln+1.5, Baseline +1.5 mmol/l.

**TABLE S2**. (continuation)

| OBLA | | | | | | Bsln+1.5 | | | | | |
| --- | --- | --- | --- | --- | --- | --- | --- | --- | --- | --- | --- |
| **S1** | | | **S2** | | | **S1** | | | **S2** | | |
| **HR** | **VO_2_rel** | **Power** | **HR** | **VO_2_rel** | **Power** | **HR** | **VO_2_rel** | **Power** | **HR** | **VO_2_rel** | **Power** |
| 181 | 52.2 | 235.9 | 187 | 57.3 | 256.6 | 173 | 48.6 | 216.1 | 176 | 53.1 | 232.8 |
| 171 | 31.6 | 89.7 | 170 | 28.5 | 72.0 | 156 | 26.1 | 64.3 | 161 | 25.2 | 53.1 |
| 138 | 34.8 | 163.2 | 139 | 35.0 | 171.9 | 131 | 32.0 | 147.5 | 126 | 30.3 | 145.6 |
| 156 | 30.4 | 128.1 | 148 | 28.2 | 126.2 | 133 | 22.6 | 87.9 | 129 | 22.7 | 91.9 |
| 160 | 34.9 | 143.5 | 159 | 34.8 | 155.1 | 142 | 29.8 | 113.3 | 143 | 30.3 | 128.3 |
| 152 | 27.3 | 95.4 | 148 | 23.0 | 77.2 | 128 | 20.8 | 69.1 | 142 | 21.3 | 69.7 |
| 154 | 47.0 | 197.6 | 151 | 44.4 | 192.9 | 145 | 43.5 | 180.3 | 143 | 41.1 | 176.0 |
| 184 | 41.1 | 207.1 | 173 | 38.5 | 198.4 | 171 | 37.2 | 183.0 | 150 | 31.8 | 158.5 |
| 163 | 42.1 | 170.3 | 148 | 35.3 | 150.0 | 149 | 35.7 | 138.4 | 140 | 32.2 | 132.9 |
| 168 | 32.9 | 151.3 | - | - | - | 149 | 27.5 | 121.3 | - | - | - |
| 154 | 46.0 | 245.5 | 163 | 48.5 | 269.7 | 143 | 41.4 | 217.9 | 150 | 43.7 | 238.3 |
| 176 | 36.8 | 191.6 | 165 | 32.1 | 172.5 | 161 | 32.8 | 164.4 | 155 | 29.6 | 154.9 |
| 173 | 33.4 | 143.3 | 171 | 34.7 | 155.8 | 161 | 30.4 | 127.2 | 163 | 32.5 | 143.1 |
| 151 | 40.4 | 209.9 | 149 | 39.9 | 215.9 | 141 | 36.9 | 187.1 | 135 | 35.1 | 183.7 |
| 168 | 40.1 | 175.9 | 160 | 38.6 | 164.3 | 160 | 37.4 | 162.0 | 148 | 34.9 | 144.8 |
| 172 | 30.7 | 183.5 | 184 | 36.8 | 213.7 | 156 | 26.5 | 152.3 | 176 | 34.1 | 194.1 |

**TABLE S3**. HRV thresholds in session 1 and session 2 with measures of HR (bpm), relative VO_2_ (ml·kg^-1^·min^-1^), and power output (W) for each participant, and maximum artifact percentage over the DFA a1 span from 1.0 to 0.5.

| **Participant number** | **HRVT1** | | | | | | **HRVT2** | | | | | | **Artifacts (%)** | |
| --- | --- | --- | --- | --- | --- | --- | --- | --- | --- | --- | --- | --- | --- | --- |
|  | **S1** | | | **S2** | | | **S1** | | | **S2** | | | **S1** | **S2** |
|  | **HR** | **VO_2_rel** | **PO** | **HR** | **VO_2_rel** | **PO** | **HR** | **VO_2_rel** | **PO** | **HR** | **VO_2_rel** | **PO** |  |  |
| **1** | 172 | 44.0 | 190.9 | 169 | 44.6 | 193.6 | 179 | 47.6 | 210.4 | 182 | 50.6 | 227.6 | 0.00 | 0.00 |
| **2** | 168 | 26.8 | 76.4 | 159 | 22.7 | 57.3 | 176 | 28.8 | 84.8 | 167 | 23.6 | 61.1 | 0.00 | 0.00 |
| **3** | 149 | 38.3 | 190.8 | 145 | 35.7 | 180.8 | 158 | 41.5 | 209.7 | 154 | 39.0 | 201.1 | 0.00 | 0.00 |
| **4** | 174 | 35.8 | 165.3 | 124 | 20.0 | 82.9 | 181 | 37.9 | 176.4 | 161 | 32.9 | 158.6 | 0.00 | 3.45 |
| **5** | 147 | 27.5 | 112.8 | 144 | 29.6 | 132.3 | 161 | 33.0 | 143.8 | 154 | 32.6 | 149.6 | 0.00 | 0.00 |
| **6** | 168 | 29.3 | 108.1 | 160 | 21.9 | 77.2 | 175 | 32.0 | 118.9 | 175 | 28.1 | 104.2 | 0.00 | 0.00 |
| **7** | 153 | 46.0 | 198.1 | 160 | 46.3 | 207.6 | 172 | 53.7 | 236.4 | 176 | 52.1 | 237.4 | 0.00 | 0.00 |
| **8** | 182 | 38.4 | 194.3 | 178 | 37.1 | 196.5 | 188 | 40.8 | 208.3 | 185 | 40.0 | 214.0 | 0.00 | 0.00 |
| **9** | 162 | 36.8 | 150.4 | 154 | 34.9 | 155.8 | 170 | 42.2 | 177.6 | 165 | 40.1 | 183.9 | 0.00 | 0.00 |
| **10** | 176 | 33.5 | 163.2 | - | - | - | 182 | 36.1 | 177.5 | - | - | - | 0.00 | - |
| **11** | 164 | 48.0 | 264.1 | 159 | 46.1 | 256.7 | 178 | 52.9 | 294.4 | 174 | 51.5 | 291.3 | 2.08 | 0.62 |
| **12** | 162 | 33.4 | 170.4 | 157 | 30.3 | 163.4 | 178 | 37.1 | 195.6 | 168 | 32.5 | 178.1 | 2.69 | 1.26 |
| **13** | 184 | 33.8 | 148.2 | 177 | 33.4 | 157.7 | 193 | 38.0 | 170.5 | 185 | 36.9 | 177.8 | 0.00 | 0.87 |
| **14** | 145 | 36.1 | 184.1 | 146 | 36.8 | 201.3 | 159 | 41.9 | 220.5 | 157 | 40.9 | 228.0 | 0.00 | 0.00 |
| **15** | 171 | 39.0 | 172.6 | 161 | 36.7 | 157.9 | 177 | 42.0 | 188.6 | 171 | 40.1 | 176.0 | 2.25 | 0.00 |
| **16** | 174 | 29.4 | 176.6 | 169 | 30.8 | 176.1 | 183 | 32.8 | 202.0 | 179 | 34.2 | 200.3 | 0.00 | 1.18 |

S1, session 1; S2, session 2; HR, heart rate; VO_2_rel, relative oxygen uptake; PO, power output; HRVT1, first heart rate variability threshold; HRVT2, second heart rate variability threshold.
